# Editorial: Resignation and strategic retention: shaping the future workforce

**DOI:** 10.3389/fpsyg.2026.1865793

**Published:** 2026-05-28

**Authors:** Linda Ronnie, Nelesh Dhanpat, Sumari O'Neil

**Affiliations:** 1 School of Management Studies, University of Cape Town, Cape Town, South Africa; 2 Department of Industrial Psychology and People Management, University of Johannesburg, Johannesburg, South Africa; 3 Department of Human Resource Management, University of Pretoria, Pretoria, South Africa

**Keywords:** employee retention, leadership, organizational well-being, post-pandemic work, resignation, talent management, workforce psychology

## Background

The COVID-19 pandemic accelerated structural changes to how work is organized, where it takes place, and what workers expect from their employers. Debates around the Great Resignation, quiet quitting, and hybrid arrangements have created urgent demand for research that moves beyond descriptive accounts to examine the mechanisms through which employee retention is shaped, sustained, and compromised. This editorial introduces a Research Topic of articles that collectively address the retention challenge from multiple vantage points. United by a shared concern with the conditions under which employees choose to remain engaged and what employers, leaders, and practitioners can do to influence those conditions; these studies converge on a core insight: retention is not primarily a matter of compensation or contractual terms, but of relational quality, psychological safety, and organizational integrity

## Overview of the Research Topic

This Research Topic brings together 17 articles authored by 56 researchers spanning institutions and continents. The Research Topic draws on a wide range of methodological approaches, including moderated mediation models, latent profile analysis, mixed methods design, instrument validation studies, and scoping reviews. This breadth of contribution, both geographically and methodologically, reflects the global and multidisciplinary nature of the retention challenge itself. The research is organized into five thematic clusters. Several studies speak to more than one theme, and readers are encouraged to move across clusters rather than treat them as discrete compartments.

## Leadership and its consequences for retention

The largest cluster addresses how leadership behavior shapes conditions under which employees choose to stay or leave. Zhang, Wang, Jiang, et al. examine exploitative leadership through Conservation of Resources (COR) theory, demonstrating that self-control depletion mediates its effects on turnover intentions and workplace procrastination, with perceived organizational support functioning as a protective buffer. Chen et al. present complementary findings from Chinese technology professionals, confirming that destructive leadership depletes psychological resources and accelerates burnout and exit intent, while identifying regulatory emotional self-efficacy (RESE) as a moderating factor—suggesting that resilience-building may usefully complement structural interventions.

Zhang and Wang introduce an important complication. Drawing on COR theory and data from Generation Z knowledge workers in Chinese SMEs, they reveal that empowering leadership is a double-edged sword: it simultaneously raises retention by enhancing perceived organizational support and lowers it by exacerbating emotional exhaustion. The direction of effects depends on employees' goal orientations, meaning that indiscriminate empowerment may burden the very employees leaders seek to support. Xu et al. turn to primary healthcare workers; a group whose disengagement carries direct public health consequences. Temporal leadership reduces quiet quitting by building time management competencies and enriching the work-family interface, with organizational communication amplifying these effects. José et al. close this cluster by demonstrating that transformational leadership positively affects perceived quality of life, but that this relationship is moderated by work regime (remote, hybrid, or in-person), underscoring that effective leadership cannot be context-agnostic.

## Employee wellbeing, burnout, and emotional resources

Three studies attend to the psychological resources employees draw upon to remain engaged, and the conditions that deplete them. Freedman et al. present striking longitudinal evidence of what they term an “Emotional Recession”: a global 5.79% decline in emotional intelligence (EQ) across 28,000 adults in 166 countries between 2019 and 2024. The sharpest falls occurred in drive-related competencies including intrinsic motivation and optimism. Individuals with higher EQ were more than ten times more likely to report strong life outcomes. The implications for workforce engagement, resilience, and retention are substantial.

Bam and Lulema take a deliberately different approach, using narrative inquiry to trace the experiences of professional women living with systemic lupus erythematosus (SLE). Their study introduces the Continuum of Embodied Challenges, a conceptual framework illuminating how episodic chronic illness intersects with gendered and ableist workplace norms to impose extraordinary invisible labor on workers managing fluctuating capacity. The study is a timely reminder that retention scholarship must account for the structural conditions that make staying in work disproportionately costly for certain groups.

Fan et al. examine job embeddedness among specialist nurses in China, using latent profile analysis to identify three distinct subgroups: a low-embedded alienation group, a medium-embedded group, and a high-embedded identity group. Professional mission emerged as a significant predictor of higher embeddedness, alongside factors such as self-assessed health and manageable work intensity. The findings call for targeted managerial strategies based on nurses' embeddedness profiles rather than uniform retention initiatives.

## Human resource practices and organizational systems

Four studies examine retention from the perspective of formal HR practices and organizational systems, reflecting the growing recognition that individual-level interventions alone cannot address structural drivers of attrition. Alzuman et al. present the development and validation of the Organizational Health Behavior Index (OHBI), a mixed-methods instrument encompassing both quantitative subscales (covering awareness, engagement, and communication satisfaction) and qualitative subscales (covering organizational culture and employee voice). Validated across 7,548 workers in Saudi industries, the OHBI offers a theoretically grounded and psychometrically robust tool for assessing retention conditions.

Lee and Kim investigate how family-friendly policies (FFPs) and employee welfare policies (EWPs) affect quality of life, public service satisfaction, and turnover intention in the Korean public sector. Drawing on the JD-R model, they find EWP satisfaction is the more robust predictor across all outcomes, and that the retention benefits of FFPs are amplified under conditions of high job autonomy highlighting nuanced interaction effects with practical implications for designing context-sensitive HR strategy.

Saurombe investigates talent management from the perspective of employees in a South African provincial government department, using a mixed-method case study. Participants emphasized the importance of proactive, inclusive leadership and practices that enable continuous human capital development. The findings highlight unmet needs around work-life balance, skills development, and succession planning, gaps that are unlikely to be unique to the South African public sector. Zhang, Wang, Lee, et al. examine guanxi HRM practices in the Chinese context where the preferential allocation of resources and opportunities based on personal relationships. Using a three-wave survey, they find that guanxi practices are associated with increased turnover intention and reduced voice behavior, mediated by diminished job meaningfulness. Notably, ethical leadership partially mitigated these harmful effects, highlighting the boundary conditions under which informal HR practices become corrosive.

## New and evolving work contexts

Three studies address changing structural conditions in how work is found, evaluated, and performed, reflecting the increasingly non-traditional landscape of contemporary employment. Naidu et al. investigate the candidate experience of virtual interviewing, using qualitative interviews to surface both the appeal and the limitations of this now-prevalent recruitment modality. Their findings point to fairness and geographic reach as genuine strengths of virtual formats, while one-way asynchronous communication and technology barriers remain meaningful deterrents. The research equips HR practitioners with candidate-centered insight at a moment when digital recruitment is unlikely to recede.

Scheel et al. study motivation and performance in microtasking crowdsourcing environments, where the traditional employment relationship is effectively absent. Against expectation, their experimental findings reveal that autonomous motivation does not mediate the effects of job characteristics on performance or commitment. Instead, it is the mitigation of a motivation, rather than the cultivation of autonomous motivation, that drives performance and platform commitment. These findings challenge core assumptions of self-determination theory in the context of gig and platform work.

Zheng et al. offer a scoping review of job search self-efficacy (JSSE) research published between 2019 and 2025, synthesizing 22 empirical studies within the Social Cognitive Career Theory framework. Key antecedents of JSSE include career adaptability, emotional intelligence, and mentoring support. The review identifies a disciplinary imbalance toward short-term outcome measurement and over-reliance on student samples that are important methodological gaps given the rapidly shifting nature of labor markets globally.

## Trust, relational dynamics, and contextual retention factors

Two studies attend to retention within specific relational and national contexts. Ma and Zhang examine how trust congruence between subordinates' expectations of being trusted (ELT) and their perceptions of actually being trusted (PLT) shapes upward ingratiation (UI) and the mediating role of ambivalent relational identity. Using three-wave dyadic data from 330 supervisor–subordinate pairs, they find that trust misalignment generates identity conflict that drives strategic ingratiation, a form of “smiling while struggling,” with practical implications for authentic workplace relationships and psychological safety.

El Mountasser and Sahraoui provide empirical grounding from a frequently underrepresented context: private higher education institutions in Morocco. Their quantitative investigation of 196 employees identifies seven retention-relevant factors, with job satisfaction, organizational climate, and career development emerging as particularly influential. The study's actionable recommendations including personalized growth plans, regular feedback mechanisms, and competency-based training are applicable well-beyond the Moroccan context.

## Conclusion

Collectively these 17 studies resist any simple formula for retaining talent. They reveal that an organization's workforce is not a stable resource to be managed but a dynamic relational field shaped by leadership behavior, institutional structures, individual psychological resources, and evolving contexts. The most consistent finding is perhaps the most significant: the forces that drive people out of organizations are frequently invisible, relational, and cumulative, while the formal retention strategies deployed in response are often generic, reactive, and insufficiently attentive to context. The editors hope this Research Topic stimulates further research across these five thematic areas and proves useful to HR practitioners, organizational leaders, and policymakers seeking to build workplaces in which people genuinely choose to stay.

